# Feeding Pigs Without Inorganic Phosphate Supplementation from Weaning to Slaughter: Contribution to More Sustainable Swine Production

**DOI:** 10.3390/ani16182899

**Published:** 2026-09-15

**Authors:** Jakob Leskovec, Joan Tarradas, Núria Tous, Beatriz Jimenez-Moya, Marc Bagaria, Peter Ader, David Torrallardona

**Affiliations:** 1 Animal Nutrition, Institut de Recerca i Tecnologia Agroalimentàries (IRTA), Mas Bové, 43120 Constantí, Catalonia, Spain; jakob.leskovec@irta.cat (J.L.); joan.tarradas@irta.cat (J.T.); nuria.tous@irta.cat (N.T.); beatriz.jimenez@irta.cat (B.J.-M.); 2 Animal Welfare, Institut de Recerca i Tecnologia Agroalimentàries (IRTA), Veïnat de Sies, 17121 Monells, Catalonia, Spain; marc.bagaria@irta.cat; 3 BASF SE, Nutrition & Health, Carl-Bosch-Str. 38, 67056 Ludwigshafen am Rhein, Germany; peter.ader@basf.com

**Keywords:** pig production, phytase, inorganic phosphate-free feeding, weaning, bone characteristics, sustainability

## Abstract

The viability of raising pigs from weaning to slaughter with inorganic phosphorus (iP)-free diets using a hybrid 6-phytase was tested in two trials. Three experimental diets were formulated: T1, a control diet with monocalcium phosphate (MCP); T2, a diet without MCP + phytase (P and Ca phytase matrix formulation); and T3, a diet without MCP + phytase (P, Ca + extra nutrients in a phytase matrix formulation). Rapeseed and sunflower meal were used in addition to soybean meal as protein sources, in order to increase the phytate-P content. Performance was monitored from weaning to slaughter, and bones were sampled at the slaughterhouse to test for bone mineralization. In the second trial, gait scores were also assessed at 75, 110, and 134 days. Weight gain was not significantly affected by treatment in either trial. However, in Trial 1, males receiving T2 had a reduced overall feed efficiency than males receiving T1, whereas in Trial 2, a reduced overall feed efficiency was observed for T3 relative to T2 (*p* < 0.05), probably due to the extra nutrient reduction. No differences in bone mineralization or gait score were observed. In conclusion, it is possible to raise pigs without iP by using phytase in feeds containing phytate-P. This may contribute to more sustainable pig production with reduced P excretion and lower usage of rock phosphate, a limited resource.

## Simple Summary

Phosphorus (P) is an essential element involved in physiological pathways, energy metabolism, and bone formation, among other vital functions. The P in vegetable ingredients in pig feeds is primarily in the form of phytate-P, which has very low availability and cannot satisfy monogastric animals’ dietary requirements. Thus, P in pig feeds is mainly provided by inorganic sources, which are highly digestible but non-renewable with rather limited natural reserves. This study tests the viability of raising pigs without using inorganic phosphorus (iP) from weaning to slaughter, instead using a hybrid 6-phytase in phytate-P rich diets. Our results show that, by adequately dosing phytase in feeds containing sufficient phytate-P, it is possible to raise pigs with iP-free diets without negatively influencing performance and bone parameters. This approach may substantially contribute to more sustainable pig production by reducing P emissions and the usage of rock phosphate, a limited resource.

## 1. Introduction

Phosphorus is an essential element that plays many biological roles and is involved in several physiological pathways, including growth; energy metabolism, as it is a component of adenosine triphosphate (ATP); and the formation of bones and teeth [1]. As P is expensive and a scarce element in nature, we aim to minimize the addition of inorganic sources in feeds. The reduction and limited usage of inorganic P sources normally do not affect feed intake and growth, although they can deteriorate P concentrations in bones [2]. This is also reflected in nutrition requirements, as higher contents are needed to maximize bone mineralization and strength than to maximize feed intake and ADG [1,3]. Thus, limiting P supply can negatively affect animals’ welfare by causing lameness and other locomotion problems [4].

Phosphorus in feed is mainly provided through inorganic sources such as monocalcium and dicalcium phosphates, which are highly digestible, although they have the inconvenience of being non-renewable resources with very limited natural reserves, leading to high and rather variable costs. Phosphorus is also present in the vegetable ingredients used for feed manufacturing, primarily as a phytic acid [5]. In general, although phytic acid (PA; dihydrogenphosphate ester of inositol) is present in most plant-based feedstuffs, it is practically indigestible for monogastric animals [6,7], making it one of the most important antinutritive components in feeds. Phytic acid can bind di-trivalent cations into stable complexes, like Ca, Mg, Fe, Zn, Cu, and Mn, making them less digestible in monogastric animals [6]. Furthermore, phosphorus bound in PA represents a significant economic loss and environmental issue, since phosphorus minerals are predicted to be depleted within the next few centuries [8]. Phytate can also inhibit the biological function of feed proteins, affect their solubility, and impair digestive enzymes, thus negatively impacting the digestion of nutrients [9]. Phytate forms complexes with carbohydrates and lipids, thus reducing the amount of energy and nutrients utilized from feedstuff [9]. The formation and stability of the formed complexes are determined by the pH, ionic strength, supporting electrolytes, temperature, nature, and concentrations of binding substances [10]. All of these factors affect the interactions of phytate with other substances present in the gastrointestinal tract (GI).

It is common practice to supplement pig feed with microbial phytase to release nutrients from complexes with phytate [11]. Released phytate-P can be considered a phosphorus supplement, while other nutrients contribute to a so-called extra phosphoric effect [11]. In general, adding microbial phytases increases the digestibility of many nutrients and the utilization of energy, thus affecting performance parameters. Phytate degradation is very complex and is determined by the source and type of dietary phytase, the feed composition (phytate source and feed-derived phytases), phytases excreted by the GI’s microbiota, and gastrointestinal conditions (passage rate, pH, etc.). To a lesser extent, the animal’s sex and growth rate, feed production parameters (heat, water content, pressure, friction, etc.), and endogenous phytase are influencing factors, where the latter has been mainly reported for poultry [11,12,13,14,15,16].

Therefore, the aim of the present study was to evaluate the dietary inclusion of a hybrid 6-phytase to test the viability of raising pigs for fattening, from weaning to slaughter, using diets without inorganic phosphorus. Two different dietary conditions were applied and compared to a control group fed a standard diet; namely, (i) feed formulated without an inorganic phosphorus source and supplemented with phytase, and (ii) feed with reduced levels of energy and nutrients (other than Ca and P) formulated according to the Natuphos E matrix (BASF SE, Ludwigshafen, Germany) for nutrients and energy and supplemented with phytase. To evaluate the viability of replacing inorganic phosphate with an adequate dose of phytase, performance parameters and bone mineralization were monitored.

## 2. Materials and Methods

### 2.1. Experimental Design, Animals, and Diets

Two trials were conducted, each with 72 newly weaned pigs ([Large white × Landrace] × Pietrain; mixed 50% entire males and 50% females; ca. 26 days of age) with body weights of 7.41 ± 0.40 and 7.80 ± 0.37 kg for Trials 1 and 2, respectively. In each trial, piglets were allocated into twelve blocks (six for each sex) according to their initial body weight. Each block consisted of three pens with two animals per pen.

For each trial, three experimental diets were formulated to meet or exceed the minimum nutrient requirements according to FEDNA [17]. Treatments consisted of a positive control diet including monocalcium phosphate (MCP; T1); a diet without MCP, supplemented with phytase and formulated using a reduced phytase matrix for P and Ca (T2); and another diet without MCP, supplemented with phytase and formulated using an extended phytase matrix for nutrients and energy, supplied by BASF SE (T3). A bacterial 6-phytase (Natuphos E, BASF, SE, Germany) assembled from sequences of various phytase-producing bacteria (i.e., *Hafnia* ssp., *Yersinia* ssp., and *Buttauxiella* ssp.) and produced by fermentation in *Aspergillus niger* was used to formulate the diets without inorganic phosphates. Rapeseed and sunflower meal were included in the diets as the main plant-based protein sources, in addition to soybean meal, in order to adjust the phytate-P content.

Table 1 shows the feeding programs applied and the doses of phytase administered in each trial.

In both trials, for the T2 formulation, only MCP was completely removed from the diet. Conversely, for the T3 formulation, in addition to complete removal of MCP, all other nutrients and energy were reduced according to the Natuphos E matrix (Table 2). In the first trial, no experimental treatments were applied during the first 7 days (pre-starter-1 phase), and all animals received an identical MCP-free diet with 2000 FTU/kg. In the second trial, a matrix value of 2000 FTU/kg was used when 3000 FTU/kg was added to the pre-starter diet.

The feed was presented in pelleted form (except pre-starter-1, which was presented in mash form) and offered ad libitum. Feed enzyme preparations were added to the feed mixer before pelleting, which was conducted at <70 °C.

The estimated composition of the key nutrients used to formulate the different experimental feeds is presented in Table 3 and Table 4. The composition of the experimental diets with their estimated and analyzed nutrient contents are presented in Supplementary Tables S1–S4. Water was provided ad libitum to all the animals throughout the experimental periods.

### 2.2. Performance Evaluation and Feed Analysis

Individual BW and feed intake for each pen were recorded at days 0, 7, 21, 43, 74, 112, and 144 and at days 0, 14, 41, 71, 116, and 138 for Trials 1 and 2, respectively, which coincided with every change in dietary specification. The pen’s average initial and final live body weight (BW), average daily weight gain (ADG), average daily feed intake (ADFI), and average gain-to-feed ratio (GFR) were calculated for each period and trial.

Proximate nutrient analyses of feeds were conducted according to [19]. Total calcium and total phosphorous were analyzed through inductively coupled plasma-optical emission spectrometry (ICP-OES) using a SPECTRO ARCOS FHS16 (SPECTRO Analytical Instruments GmbH, Kleve, Germany), according to method 984.27 [19]. Phytate P was determined using the method described by Haug and Lantzsch [20], and in-feed phytase activity was determined according to [21].

### 2.3. Bone Mineralization and Gait Score

At the end of each trial, pigs were taken to the slaughterhouse. The front left hoof of each pig was sampled and kept frozen until analysis. The corresponding left Os metacarpale III was obtained and analyzed for dry matter (method 934.01) and ash contents (method 942.05) [19].

In Trial 2, lameness was individually assessed in all pigs at days 75, 110, and 134 according to three score categories (0 = normal gait; 1 = the animal is severely lame; and 2 = there is no weight-bearing on the affected limb, or the animal is unable to walk).

### 2.4. Statistical Analysis

The effects of dietary treatment on performance and bone mineralization were analyzed via ANOVA using the GLM procedure of SAS (SAS Inst. Inc., Cary, NC, USA, version 9.4). A randomized block design was used with the initial weight and pen location as block criteria. For the statistical analysis, the effects of block, sex, treatment, and sex-by-treatment interaction were included in the model, and the pen was used as the experimental unit. The mathematical model used was Yijk = μ + βi + Sj + τk + (S × τ)jk + εijk, where Yijk is the value for the observation of the block group (i), sex (j), and treatment (k); μ is the general mean of all observations; βi is the effect of the block group (i); Sj is the effect of sex (j); τk is the effect of treatment (k); (S × τ)jk is the effect of the interaction between sex (j) and treatment (k); and εijk is the unexplained random error. The data in the tables are presented as least-square means, and the Tukey adjustment was applied to perform multiple comparisons in case of significant F values for the sex-by-treatment interaction.

## 3. Results

### 3.1. Performance

**Figure 1:**
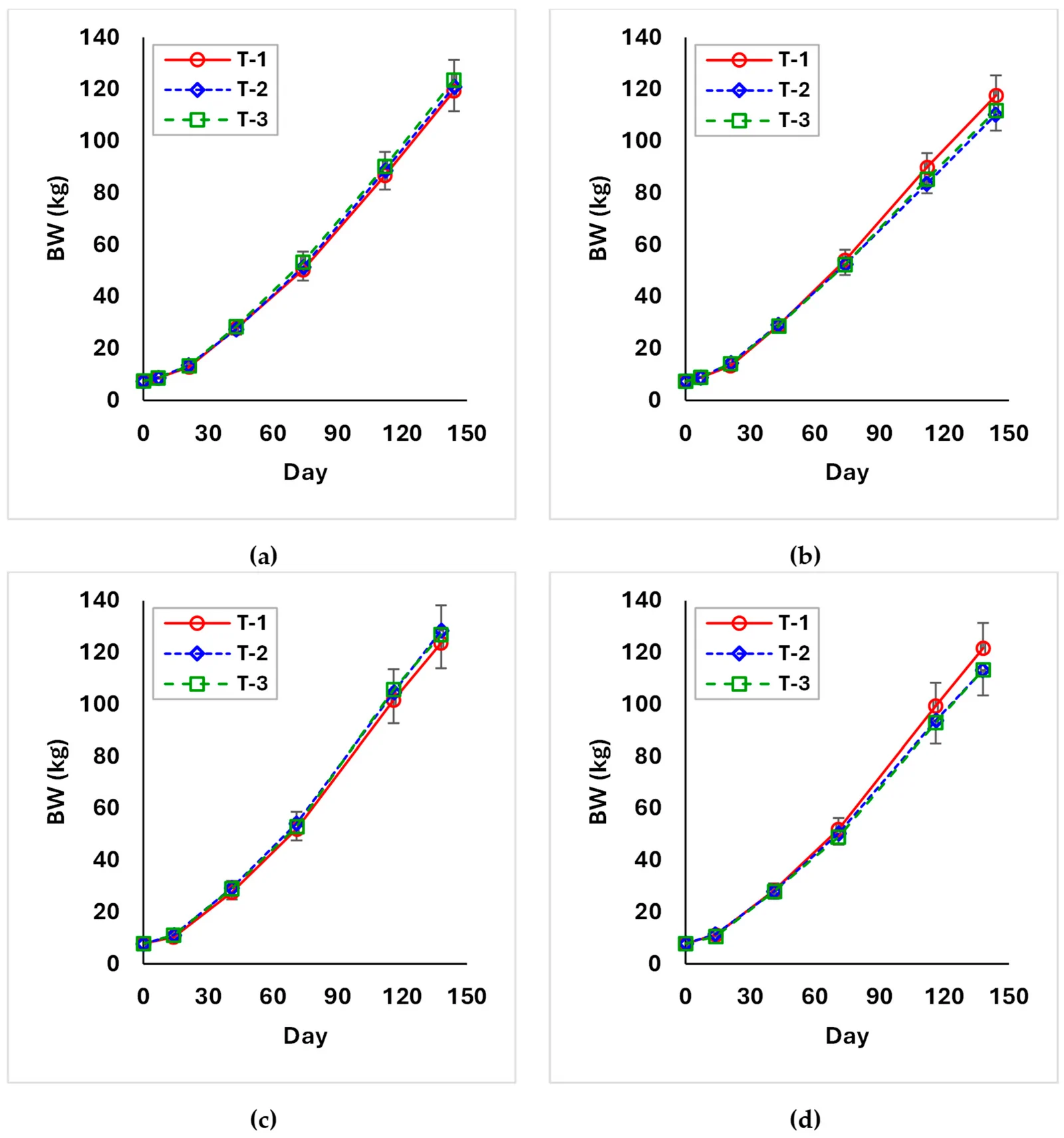
Body weight. (**a**) Trial 1—males; (**b**) Trial 1—females; (**c**) Trial 2—males; and (**d**) Trial 2—females.

The development of body weight in male and female pigs from both trials is presented in Figure 1. Pigs’ body weight was not significantly affected by dietary treatment compared to T1, and as expected, female pigs had significantly lower final weights (*p* < 0.01). However, despite not observing treatment-by-sex interactions (*p* > 0.1), from a numerical point of view, females (but not males) in both trials appeared to have slightly lower body weights during the second growing phase and the finisher phase when fed the experimental diets (T2 and T3) (Figure 1b,d).

The performance results of Trial 1 are presented in Table 5. No differences among treatments were observed for average daily weight gain in any of the periods considered or the total period of the study. From days 7 to 21, females tended to have higher weight gain than males (*p* = 0.07). However, from day 74 onward, females gained significantly less weight compared with males, and this effect was also observed when considering the overall experimental period (*p* < 0.01). From days 43 to 74, a tendency for an interaction between sex and treatment was observed (*p* = 0.08), as females in the control group (T1) gained numerically more weight than males undergoing the same treatment, while the opposite was observed in T3. Nevertheless, differences in weight gain between T2 or T3 and the control (T1) could not be found for male nor female pigs.

Regarding average daily feed intake, treatment was observed to have a significant effect from days 7 to 21 in which the animals receiving T2 had a higher feed intake than those in the control group (*p* < 0.05). In the periods of 0 to 7 and 7 to 21 days, females consumed more feed than the corresponding males (*p* < 0.05), and the same trend (*p* < 0.1) was also observed for the period of 21 to 43 days. Interactions between sex and treatment were observed for the period from 43 to 74 days in which females in the T1 group, but not T2 and T3, ate more than the corresponding males, and for the periods of 74 to 112 days, 112 to 144 days, and the whole trial (0 to 144 days), where females in T2, but not T1 and T3, ate less than the corresponding males.

Regarding the gain-to-feed ratio, treatment was observed to have a significant effect within days 21 to 43 in which the animals receiving T3 had worse feed efficiency than those in the control group (*p* < 0.01). Males had better feed efficiency than females in that period (*p* < 0.01), and the same trend was observed from days 74 to 112 (*p* = 0.05). An interaction (*p* < 0.01) between sex and treatment was also observed for the gain-to-feed ratio from days 112 to 144 and for the whole trial period (0–144 days). From days 112 to 144 and over the whole study period (0 to 144 days), females in the T1 and T3 groups had significantly lower gain-to-feed ratios than males in the corresponding treatments, whereas differences between sexes were not observed in T2. Overall, in males, the gain-to-feed ratio of T1 was not achieved through T2, whereas no significant differences among treatments were observed in female pigs or for any of the sexes between T3 and T1.

The growth performance results from Trial 2 are presented in Table 6. No effects of treatments on weight gain were observed in any of the periods considered or in the total study period. For the periods of 41 to 71 and 71 to 116 days, and for the overall experimental period, males gained significantly more weight compared with females (*p* < 0.05), while a tendency for an interaction between sex and treatment was observed from 116 to 138 days (*p* = 0.07), where males had higher weight gain than females for T2 but not for T1 and T3.

No effects of treatments nor interactions between treatment and sex were observed for average daily feed intake in any of the periods considered or in the whole study (0 to 138 days). However, from day 71 onward, males had significantly higher feed intake than females, and this effect was also observed when considering the overall experimental period (*p* < 0.05).

Significant effects of treatment on gain-to-feed ratio were observed from days 116 to 138 and for the overall study period (*p* < 0.05) as animals receiving T2 had better feed efficiency than those receiving T3, while no difference on that parameter was found between the two treatments (T2 and T3) and the control (T1). From days 14 to 41, a trend was observed in which animals receiving T3 tended to grow less efficiently than those receiving T1 (*p* = 0.06). Males had better feed efficiency (*p* < 0.05) than females in the periods of 41 to 71 and 71 to 166 days and in the overall study period (0 to 138 days). No interactions between sex and treatment were observed in any period for this parameter.

### 3.2. Bone Mineralization and Gait Score

The results for bone mineralization for Trials 1 and 2 are shown in Table 7. No statistically significant effects of treatment nor interaction between sex and treatment were observed for any of the parameters considered. However, in both trials, the left Os metacarpale III of females had lower dry weight (−2.15 and −3.03 g/bone in Trials 1 and 2, respectively) and ash weight (−0.53 and −0.73 g/bone in Trials 1 and 2, respectively) than males, but the ash percentage was higher in females than in males (by 2.87 and 2.86% units in Trials 1 and 2, respectively).

**Figure 2:**
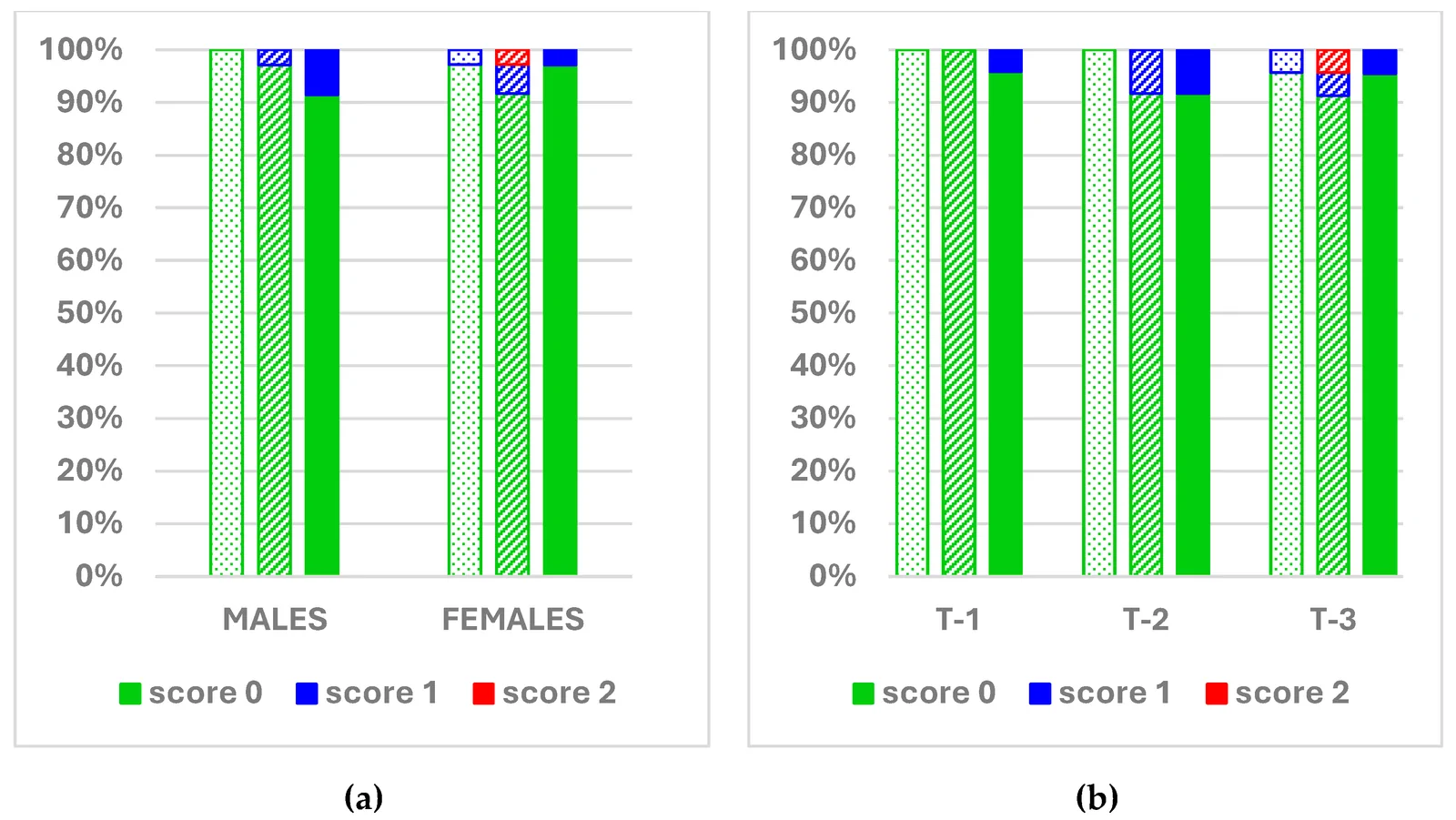
Gait score frequencies at days 75 (dotted bars), 110 (lined bars), and 134 (solid bars) of Trial 2 (**a**) by sex and (**b**) by treatment. Chi square *p* values for sex effect at days 75, 110, and 134 were 1.000, 1.000, and 0.614, respectively; those for treatment effect were 0.324, 0.311, and 1.000, respectively.

Gait score frequencies at days 75, 110, and 134 of Trial 2 are presented in Figure 2. More than 90% of the animals in the experiment had a gait score of 0 (normal gait). No effects were observed for sex or treatment.

## 4. Discussion

Due to scarcity and high cost of phosphorus, the pig industry must find alternatives to replace inorganic sources. The main alternative is using microbial phytases, which were developed in the late 1980s [22]. Microbial phytases were constantly improved with research focusing on matrix values, optimal activity conditions, and other important conditions to increase their efficacy [23]. Therefore, including even low levels of microbial phytase hydrolyses up to 60% of phytate, while higher doses reach up to 90% [23].

This allows for pigs to be raised without inorganic phosphorus [24,25], although concerns arise regarding the use of phytase and phytate-P as the sole dietary phosphorus sources. Growing pigs completely without inorganic sources of phosphorous is challenging and yields variable results due to differences in phytate hydrolysis, pH changes in the intestinal tract, and the optimal range of activity, retention time in the GIT, age of animals, phytate properties, matrix values, phytate variability, and interactions with other nutrients (Ca, Zn, Fe, proteins, and amino acids) [23]. Therefore, even though total replacement of inorganic P with phytase is possible, caution is needed because there is no safety margin in the case of insufficient total phytate-P, phytate degradation, and P metabolism.

### 4.1. Performance Parameters

In both trials, no statistically significant differences in pigs’ body weights were observed between the dietary treatments. Under our study’s conditions, pigs were raised from weaning to slaughter without dietary iP sources, but with adequate dosing of a modern hybrid 6-phytase; notably, this did not result in any negative impacts on the animals, when compared to the positive controls (T1). Our study is similar to other pig trials, where a sufficient amount of phytase supplementation, together with adequate levels of phytic phosphorus, yielded results comparable to those obtained using feeds with iP [25]. In addition, the absence of significant differences between T2 and T3 in Trial 1 and up to the end of the grower-2 phase in Trial 2 supports the extra-phosphoric effect of phytase [11]. On the other hand, the observed reduction in gain-to-feed ratio for T3, relative to T2, in the finishing phase (which also affected the overall period) of Trial 2 suggests that phytase was not able to compensate for the extra reduction in energy and nutrients applied during that particular phase. Although not statistically significant, female animals in both trials had numerically lower body weights under both iP-free experimental treatments (T2 and T3) toward the end of the trial (after day 74 or 71, respectively). This could be due to differences in nutrient requirements between females and males, especially in the finishing period when females need less protein and more energy per kg of BW than males [26]. When using phytase as the only source of P, nutrient requirements should be somewhat adapted to sex differences. The reduction in feed efficiency observed in Trial 1 for T2 compared with T1, which occurred in males but not in females, may support this. Another explanation might be that digestion kinetics could be different between sexes, especially when coupled with phytases and inorganic P-free feed. There is some evidence that sex has substantial effects on intestinal tract relative weights, which are reported to be higher in male pigs. Consequently, higher feed intake and nutrient metabolism are described for barrows compared to gilts [27]. Some other gastrointestinal differences, such as villus height, intestinal muscular layers, and microbial composition, can also likely affect digestion kinetics and metabolism as well as the efficacy of a dietary phytase. Those differences were generally reflected in weight gain and feed intake at the end of both trials, where females had lower weight gain than males. For that reason, the concept of inorganic phosphate-free feeding might require different strategies for precision feeding of both sexes. Under the conditions of our study, it can be concluded that each pig in the T2 and T3 groups consumed roughly 1350 and 1400 g less MCP, relative to T1, in Trials 1 and 2, respectively.

### 4.2. Bone Mineralization and Gait Score

Phytase inclusion and matrix implementation prevented any negative impact of removing MCP on bone mineralization, measured as dry weight of the bone, ash percentage, and total amount of ash in the Os metacarpale III in any of the two trials. This is in line with other trials involving weaned piglets, where reduced dietary levels of iP did not affect bone mineralization when sufficient phytase was added to the diets [28,29]. Similar results were observed in growing pigs, where phytase restored bone ash when used in marginally deficient diets [30]. Regarding P supply for sufficient growth, the results of this study suggest that phytase supplementation may replace inorganic phosphorus sources when dietary phytate-P content and phytase inclusion levels are appropriately managed under the conditions evaluated in this study. Additionally, some trials involving finishing pigs detected lower bone mineralization throughout the growing and finishing periods when pigs were fed a phytase-supplemented iP-free diet compared to diets containing both iP and phytase [31]. Therefore, the importance of sufficient phytate-P and phytase dose level, as well as phytase’s efficiency, is underlined again. The differences in bone mineralization between males and females could be expected and are consistent with the previous literature showing that males deposit more minerals than females [32], though interactions between sex and phytase inclusion have not been described.

Proper bone growth and mineralization contribute to appropriate locomotor function in animals. The lack of differences in mineralization of the bones was also reflected in the gait score, where no statistically significant differences were detected among groups. These findings align with a previous study in which supplementing a P-deficient diet with 500 FTU of phytase/kg improved the mechanical properties of femurs in finishing pigs [33]; this observation may correlate with the variations in gait scores. To the best of our knowledge, there is no publication reporting in-depth research on the potential correlation between mechanical properties of bones of the leg and gait score.

## 5. Conclusions

The results of the present study indicated that, under the experimental conditions evaluated, pigs can be raised with total replacement of inorganic phosphorus by incorporating phytase into diets containing sufficient phytate-P throughout the weaning-to-finishing period. While performance parameters were generally unaffected, our findings suggest that considering the different nutrient and energy requirements of each sex may be advisable when formulating feeds and determining phytase dosages. Further investigations into more detailed recommendations are therefore required. Bone mineralization was not significantly impacted by the absence of iP, supporting the potential of this phytase as an effective replacement under the experimental conditions evaluated. In summary, this strategy offers promising opportunities for more sustainable pig production, primarily by reducing P consumption (and, likely, excretion) and lowering the demand for rock phosphate, a finite resource. In certain regions, utilizing local plant-based protein sources like sunflower and rapeseed meals, which contain higher phytate-P levels than soybean meal, might offer an additional advantage.

## Figures and Tables

**Table 1:** Feeding programs and doses of phytase per kg of feed administered.

Trial 1					Trial 2				
	Phytase (FTU/kg ^1^)			Days PW		Phytase (FTU/kg)			Days PW
Phase	T1	T2	T3		Phase	T1	T2	T3	
Pre-starter-1 ^2^	-	-	-	0–7					
Pre-starter-2	0	2000	2000	7–21	Pre-starter	0	3000	3000	0–14
Starter	0	2000	2000	21–43	Starter	0	2000	2000	14–41
Grower-1	0	1000	1000	43–74	Grower-1	0	1000	1000	41–71
Grower-2	0	500	500	74–112	Grower-2	0	500	500	71–116
Finisher	0	300	300	112–144	Finisher	0	500	500	116–138

^1^ FTU: Phytase activity analyzed according to [18]. ^2^ Includes 2000 FTU Natuphos E/kg (no experimental treatments were applied during this phase). T1: Positive control with MCP; T2: negative control with phytase; T3: negative control with phytase and E matrix.

**Table 2:** Matrices of applied nutrient contribution of the different doses of phytase (FTU/kg feed) used to formulate the diets.

Nutrient	300 FTU	500 FTU	1000 FTU	2000 FTU
Reduced matrix (T2 & T3)				
Total P (g/kg feed)	1.00	1.40	1.90	2.15
Digestible P (g/kg feed)	0.80	1.12	1.52	1.72
Total calcium (g/kg feed)	1.00	1.40	1.90	2.15
Extended matrix (only T3)				
Energy (kcal ME/kg feed)	37.1	51.9	70.5	71.3
Crude protein (g/kg feed)	2.0	2.8	3.8	4.3
SID lysine (g/kg feed)	0.080	0.112	0.152	0.172
SID methionine (g/kg feed)	0.025	0.035	0.048	0.054
SID Met + Cys (g/kg feed)	0.055	0.077	0.105	0.118
SID isoleucine (g/kg feed)	0.050	0.070	0.095	0.108
SID threonine (g/kg feed)	0.050	0.070	0.095	0.108
SID tryptophan (g/kg feed)	0.030	0.042	0.057	0.065
SID valine (g/kg feed)	0.040	0.056	0.076	0.086
Zinc (g/kg feed)	0.032	0.045	0.061	0.069

**Table 3:** Estimated nutrient composition ^1^ of diets for the pre-starter and starter phases of Trial 1.

	Pre-starter-1 (0–7 days)		Pre-starter-2 (7–21 days)						Starter (21–43 days)		
			T1		T2		T3		T1	T2	T3
Energy (kcal ME/kg)	3285		3285		3285		3214 (3285)		3280	3280	3209 (3280)
SID Lysine (g/kg feed)	12.8		12.8		12.8		12.6 (12.8)		12.0	12.0	11.8 (12.0)
Total Ca (g/kg feed)	7.00		7.00		4.85 (7.00)		4.85 (7.00)		7.30	5.15 (7.30)	5.15 (7.30)
Total P (g/kg feed)	6.20		6.88		5.06 (7.21)		5.06 (7.21)		6.49	4.69 (6.84)	4.69 (6.84)
Total Ca:total P ratio	1.13		1.02		0.96 (0.97)		0.96 (0.97)		1.12	1.10 (1.10)	1.10 (1.07)
Digestible P (g/kg feed)	3.95		3.80		2.08 (3.80)		2.08 (3.80)		3.30	1.58 (3.30)	1.58 (3.30)
Phytate P (g/kg feed)	1.25		2.46		2.50		2.51		2.69	2.74	2.75
	Grower-1 (43–74 days)				Grower-2 (74–112 days)				Finisher (112–144 days)		
	T1	T2		T3	T1	T2		T3	T1	T2	T3
Energy (kcal ME/kg)	3180	3180		3173 (3180)	3175	3175		3170 (3175)	3150	3150	3146 (3150)
SID Lysine (g/kg feed)	8.90	8.90		8.75 (8.90)	7.70	7.70		7.59 (7.70)	6.30	6.30	6.22 (6.30)
Total Ca (g/kg feed)	6.70	4.80 (6.70)		4.80 (6.70)	6.50	5.10 (6.50)		5.10 (6.50)	5.90	4.90 (5.90)	4.90 (5.90)
Total P (g/kg feed)	6.05	4.43 (6.33)		4.46 (6.36)	5.94	4.74 (6.14)		4.76 (6.16)	5.81	4.97 (5.97)	4.97 (5.97)
Total Ca:total P ratio	1.11	1.08 (1.06)		1.08 (1.05)	1.09	1.08 (1.08)		1.07 (1.06)	1.02	0.99 (0.99)	0.99 (0.99)
Digestible P (g/kg feed)	2.80	1.28 (2.80)		1.29 (2.81)	2.50	1.38 (2.50)		1.39 (2.51)	2.30	1.50 (2.30)	1.50 (2.30)
Phytate P (g/kg feed)	2.81	2.84		2.87	3.02	3.05		3.06	3.02	3.05	3.05

^1^ Values in brackets include the expected nutrient contribution from Natuphos E 10000 G according to Table 2.

**Table 4:** Estimated nutrient composition ^1^ of diets for the pre-starter and starter phases of Trial 2.

	Pre-starter (0–14 days)						Starter (14–41 days)					
	T1		T2		T3		T1		T2		T3	
Energy (kcal ME/kg)	3285		3285		3214 (3285)		3280		3280		3209 (3280)	
SID Lysine (g/kg feed)	12.8		12.8		12.6 (12.8)		12.0		12.0		11.8 (12.0)	
Total Ca (g/kg feed)	7.00		4.85 (7.00)		4.85 (7.00)		7.30		5.15 (7.30)		5.15 (7.30)	
Total P (g/kg feed)	6.68		4.85 (7.00)		4.86 (7.01)		6.46		4.66 (6.81)		4.70 (6.85)	
Total Ca:total P ratio	1.05		1.00 (1.00)		1.00 (1.00)		1.13		1.11 (1.11)		1.10 (1.07)	
Digestible P (g/kg feed)	3.80		2.10 (3.82)		2.10 (3.82)		3.30		1.58 (3.30)		1.64 (3.36)	
Phytate P (g/kg feed)	2.32		2.35		2.37		2.75		2.77		2.80	
	Grower-1 (41–71 days)				Grower-2 (71–116 days)				Finisher (116–138 days)			
	T1	T2		T3	T1	T2		T3	T1	T2		T3
Energy (kcal ME/kg)	3180	3180		3110 (3180)	3175	3175		3123 (3175)	3150	3150		3098 (3150)
SID Lysine (g/kg feed)	8.90	8.90		8.75 (8.90)	7.70	7.70		7.59 (7.70)	6.30	6.30		6.19 (6.30)
Total Ca (g/kg feed)	6.70	4.80 (6.70)		4.80 (6.70)	6.50	5.10 (6.50)		5.10 (6.50)	5.90	4.00 (5.90)		4.00 (5.90)
Total P (g/kg feed)	6.06	4.48 (6.38)		4.52 (6.42)	5.95	4.67 (6.07)		4.83 (6.23)	5.35	4.28 (5.68)		4.30 (5.70)
Total Ca:total P ratio	1.11	1.07 (1.05)		1.06 (1.04)	1.09	1.09 (1.09)		1.06 (1.04)	1.10	0.93 (1.04)		0.93 (1.04)
Digestible P (g/kg feed)	2.80	1.33 (2.85)		1.33 (2.85)	2.50	1.38 (2.50)		1.44 (2.56)	2.30	1.30 (2.42)		1.31 (2.43)
Phytate P (g/kg feed)	2.79	2.82		2.83	2.97	2.99		3.01	2.60	2.61		2.62

^1^ Values in brackets include the expected nutrient contribution from Natuphos E 10000 G according to Table 2.

**Table 5:** Performance parameters of Trial 1 in pigs from 0 to 144 days.

Item	Period	Sex	T1 Positive Control (MCP)	T2 No MCP + Phytase (Red. Matrix)	T3 No MCP + Phytase (Ext. Matrix)	Root MSE	Sex Effect (Pr > F)	Treatment Effect (Pr > F)	Sex × Treatment Effect (Pr > F)
Weight gain (g/d)	0–7 d	M	176	187	176	48.9	0.216	0.944	0.780
		F	198	194	211				
	7–21 d	M	297	344	334	70.9	0.074	0.141	0.911
		F	330	402	378				
	21–43 d	M	680	632	686	64.4	0.642	0.362	0.478
		F	699	669	660				
	43–74 d	M	727	775	803	67.8	0.688	0.772	0.081
		F	815	753	765				
	74–112 d	M	960	981	972	86.8	0.004	0.372	0.154
		F	944	819	867				
	112–144 d	M	1019	1009	1039	132.0	<0.001	0.931	0.865
		F	866	833	827				
	0–144 d	M	778	789	806	54.3	0.006	0.668	0.275
		F	765	714	725				
Feed intake (g/d)	0–7 d	M	190	225	194	44.7	0.032	0.784	0.224
		F	235	223	254				
	7–21 d	M	355	414	413	60.2	0.016	0.024	0.742
		F	401	490	453				
	21–43 d	M	925	920	1029	117.1	0.096	0.160	0.814
		F	1023	993	1064				
	43–74 d	M	1454 ^y^	1518 ^xy^	1635 ^xy^	118.0	0.125	0.471	0.022
		F	1677 ^x^	1560 ^xy^	1563 ^xy^				
	74–112 d	M	2088 ^xy^	2270 ^x^	2263 ^xy^	196.8	0.074	0.590	0.048
		F	2193 ^xy^	1940 ^y^	2113 ^y^				
	112–144 d	M	2711 ^xy^	3103 ^x^	2923 ^xy^	337.8	0.171	0.916	0.046
		F	2900 ^xy^	2546 ^y^	2804 ^xy^				
	0–144 d	M	1651	1807	1805	125.9	0.411	0.531	0.021
		F	1791	1629	1736				
Gain to feed ratio	0–7 d	M	0.938	0.828	0.926	0.150	0.243	0.608	0.649
		F	0.839	0.834	0.836				
	7–21 d	M	0.821	0.819	0.812	0.085	0.494	0.814	0.810
		F	0.819	0.864	0.831				
	21–43 d	M	0.736	0.688	0.670	0.037	0.008	0.002	0.411
		F	0.684	0.675	0.625				
	43–74 d	M	0.502	0.511	0.489	0.024	0.102	0.713	0.405
		F	0.486	0.484	0.490				
	74–112 d	M	0.461	0.437	0.432	0.033	0.053	0.226	0.897
		F	0.431	0.419	0.411				
	112–144 d	M	0.373 ^a^	0.325 ^ab^	0.356 ^a^	0.029	<0.001	0.656	0.009
		F	0.298 ^b^	0.327 ^ab^	0.296 ^b^				
	0–144 d	M	0.471 ^a^	0.437 ^bc^	0.447 ^ab^	0.015	<0.001	0.042	0.005
		F	0.427 ^bc^	0.438 ^bc^	0.418 ^c^				

^abc^ For each item and period, values with different superscripts are significantly different (*p* < 0.05). ^xy^ For each item and period, values with different superscripts tend to be significantly different (*p* < 0.1).

**Table 6:** Performance parameters of Trial 2 in pigs from 0 to 138 days.

Item	Period	Sex	T1 Positive Control (MCP)	T2 No MCP + Phytase (Red. Matrix)	T3 No MCP + Phytase (Ext. Matrix)	Root MSE	Sex Effect (Pr > F)	Treatment Effect (Pr > F)	Sex × Treatment Effect (Pr > F)
Weight gain (g/d)	0–14 d	M	178	235	234	62.4	0.441	0.334	0.189
		F	239	256	201				
	14–41 d	M	640	673	667	73.9	0.246	0.854	0.640
		F	637	613	643				
	41–71 d	M	817	833	797	82.2	0.027	0.424	0.686
		F	786	748	720				
	71–116 d	M	1102	1122	1173	113.5	0.002	0.704	0.278
		F	1054	965	980				
	116–138 d	M	1001 ^ab^	1082 ^a^	964 ^ab^	111.0	0.059	0.390	0.070
		F	1015 ^ab^	886 ^b^	927 ^ab^				
	0–138 d	M	839	875	863	70.8	0.004	0.809	0.209
		F	826	764	765				
Feed intake (g/d)	0–14 d	M	273	272	304	85.5	0.902	0.870	0.316
		F	296	316	247				
	14–41 d	M	824	860	901	88.7	0.371	0.209	0.761
		F	826	808	870				
	41–71 d	M	1517	1568	1459	137.6	0.830	0.382	0.697
		F	1541	1503	1469				
	71–116 d	M	2468	2495	2661	258.5	0.029	0.517	0.296
		F	2456	2252	2321				
	116–138 d	M	2870	2915	2981	261.7	0.017	0.170	0.107
		F	2894	2470	2743				
	0–138 d	M	1781	1815	1867	148.9	0.037	0.584	0.270
		F	1789	1645	1704				
Gain to feed ratio	0–14 d	M	0.687	0.864	0.795	0.122	0.623	0.203	0.232
		F	0.801	0.805	0.801				
	14–41 d	M	0.778	0.782	0.742	0.038	0.362	0.062	0.769
		F	0.772	0.758	0.738				
	41–71 d	M	0.538	0.530	0.553	0.052	0.024	0.889	0.677
		F	0.510	0.497	0.489				
	71–116 d	M	0.447	0.449	0.441	0.021	0.015	0.753	0.969
		F	0.430	0.428	0.425				
	116–138 d	M	0.350	0.371	0.324	0.024	0.930	0.007	0.437
		F	0.352	0.359	0.338				
	0–138 d	M	0.472	0.482	0.462	0.014	0.008	0.017	0.847
		F	0.462	0.465	0.449				

^ab^ For each item and period, values with different superscripts are significantly different (*p* < 0.05).

**Table 7:** Bone characteristics of left Os metacarpale III at the end of Trials 1 and 2.

	Item	Sex	T1 Positive Control (MCP)	T2 No MCP + Phytase (Red. Matrix)	T3 No MCP + Phytase (Ext. Matrix)	Root MSE	Sex Effect (Pr > F)	Treatment Effect (Pr > F)	Sex × Treatment Effect (Pr > F)
Trial 1	Dry weight (g/bone)	M	19.0	18. 7	18.6	1.537	<0.001	0.959	0.594
		F	16.3	16.9	16.7				
	Ash (% DM)	M	40.6	40.2	41.1	2.128	<0.001	0.359	0.311
		F	44.6	43.0	42.9				
	Ash (g/bone)	M	7.71	7.69	7.87	0.672	0.006	0.972	0.828
		F	7.23	7.26	7.17				
Trial 2	Dry weight (g/bone)	M	19.0	20.0	19.1	1.866	<0.001	0.352	0.239
		F	16.9	16.4	15.7				
	Ash (% DM)	M	39. 8	39.4	40.5	0.804	<0.001	0.930	0.162
		F	43.0	42.0	43.2				
	Ash (g/bone)	M	7.44	7.88	7.78	2.567	<0.001	0.285	0.907
		F	7.24	6.87	6.79				

## Data Availability

The data presented in this study is available upon request from the corresponding author.

## Supplementary Materials

The following supporting information can be downloaded at https://www.mdpi.com/article/10.3390/ani16182899/s1, Table S1: Composition of diets for the pre-starter and starter phases of Trial 1; Table S2: Composition of diets for the grower and finisher phases of Trial 1; Table S3: Composition of diets for the pre-starter and starter phases of Trial 2; Table S4: Composition of diets for the grower and finisher phases of Trial 2.
