# Supplementary material for: Feeding Pigs Without Inorganic Phosphate Supplementation from Weaning to Slaughter: Contribution to More Sustainable Swine Production
Source: Animals (Basel). 2026 Sep 15;16(18):2899. doi: 10.3390/ani16182899 (PMC13603290; doi:10.3390/ani16182899)
Supplement: Supplementary file 1 [file animals-16-02899-s001.zip › animals-4524610-supplementary.pdf]

Article

Feeding Pigs Without Inorganic Phosphates Supplementation from Weaning to Slaughter: Contribution to a More Sustainable Swine Production

Jakob Leskovec [1], Joan Tarradas [1], Núria Tous [1], Beatriz Jiménez [1], Marc Bagaria [2], Peter Ader [3] and David Torrallardona [1,*]

Supplementary Table S1. Composition of diets for the pre-starter and starter phases of trial 1.

	Pre-starter -1 (0-7 d)	Pre-starter -2 (7-21 days)			Starter (21-43 days)		
	Common diet (all piglets)	T1 Positive Control (MCP)	T2 No MCP + Phytase (Red. Matrix)	T3 No MCP + Phytase (Ext. matrix)	T1 Positive Control (MCP)	T2 No MCP + Phytase (Red. Matrix)	T3 No MCP + Phytase (Ext. matrix)
Ingredients (g/kg feed)							
Wheat	284.1	337.1	352	380.4	316	321.7	350.2
Barley	350	200	200	200	235	235	235
Sweet milk whey	164.6	82.3	82.3	82.3	49.5	49.5	49.5
Fishmeal (70% CP)	98.9	26	26	26	-	-	-
Spray dried plasma	70	-	-	-	-	-	-
Soybean meal (48% CP)	-	168	170.4	156.7	185	187.4	173.7
Rapeseed meal	-	52.5	52.5	52.5	70	70	70
Sunflower meal (37% CP)	-	52.5	52.5	52.5	60	60	60
Animal fat	14.8	46.7	40	25.1	57	50.3	35.5
L-Lysine-HCl	0.76	4.52	4.38	4.52	4.72	4.28	4.42
L-Threonine	0.07	1.76	1.68	1.7	1.71	1.48	1.51
DL-Methionine	0.38	1.12	1.02	0.94	0.94	0.76	0.68
L-Tryptophan	0.07	0.33	0.3	0.27	0.25	0.17	0.14
L-Valine	-	0.27	0.15	0.21	0.21	-	-
Salt	-	2.39	2.4	2.4	2.5	2.5	2.51
Calcium carbonate	3.96	3.87	1.89	1.96	6.44	4.41	4.48
Monocalcium phosphate	-	8.33	-	-	8.38	-	-
Noxyfeed [1]	0.2	0.2	0.2	0.2	0.2	0.2	0.2
Vit-Min complex [2]	4	4	4	4	4	4	4
Amasil NA	8	8	8	8	8	8	8
Carbohydrase (TXU-TGU/kg) [3]	560-250	560-250	560-250	560-250	560-250	560-250	560-250
Phytase (FTU/kg) [4]	2000	-	2000	2000	-	2000	2000
Estimated nutrients (g/kg feed) [5]							
Crude Protein	203.6	202.3	204.5	200.9 (205.2)	194.5	200	196.3 (200.6)
Crude Fibre	23.1	40	40.5	40.5	44.7	45.4	45.5
Fat	38.1	64.5	58.1	43.6	73.4	66.9	52.4
Ash	53.0	55.1	45.2	44.9	54.2	44.8	44.4
Energy (kcal ME/kg)	3285	3285	3285	3214 (3285)	3280	3280	3209 (3280)
SID Lysine	12.8	12.8	12.8	12.63 (12.80)	12.00	12.00	11.83 (12.00)
SID Threonine	8.32	8.32	8.32	8.21 (8.32)	7.80	7.80	7.69 (7.80)
SID Methionine	3.84	4.33	4.28	4.17 (4.22)	3.85	3.75	3.65 (3.70)
SID Met+Cys	7.71	7.55	7.55	7.43 (7.55)	7.08	7.08	6.96 (7.08)
SID Tryptophan	2.56	2.56	2.56	2.50 (2.56)	2.40	2.40	2.34 (2.40)
Total calcium	7.00	7.00	4.85 (7.00)	4.85 (7.00)	7.30	5.15 (7.30)	5.15 (7.30)
Total phosphorous	6.20	6.88	5.06 (7.21)	5.06 (7.21)	6.49	4.69 (6.84)	4.69 (6.84)
Digestible P	3.95	3.8	2.08 (3.80)	2.08 (3.80)	3.30	1.58 (3.30)	1.58 (3.30)
Phytate P	1.25	2.46	2.50	2.51	2.69	2.74	2.75
Analysed nutrients (g/kg feed)							
Dry Matter	904	903	904	904	905	904	903
Crude Protein	188	199	203	198	191	197	195

Crude Fibre	23.8	36.4	37.7	37.8	42.9	43.1	44.7
Fat	33.1	61.4	54.7	43.1	70.2	65.3	51.1
Ash	54.1	54.5	47.4	46.6	52.6	46.2	45.1
Energy (kcal GE/kg)	3980	4188	4190	4127	4233	4253	4164
Total calcium	6.9	6.7	4.9	4.7	6.4	4.4	4.4
Total phosphorous	6.7	7.6	5.4	5.3	6.3	4.6	4.5
Phytate P	1.39	2.15	2.24	2.35	2.7	2.67	2.41
Phytase (FTU/kg)	-	-	1855	1755	-	1808	1891
% recovery	-	-	93	88	-	90	94

[1] ITPSA, Barcelona, Spain. Contains BHT+ propyl galate (56%) and citric acid (14%).

[2] Provides per kg feed: vitamin A (E 672) 10000 UI; vitamin D3 (E 671) 2000 UI; vitamin E (alfa tocopherol) 25 mg; vitamin B1 1.5 mg; vitamin B2 3.5 mg; vitamin B6 2.4 mg; vitamin B12 20 μg; vitamin K3 1.5 mg; calcium panthotenate 14 mg; nicotinic acid 20 mg; folic acid 0.5 mg; biotin 50 μg; Fe (E 1) (from $FeSO_4 \cdot H_2O$) 120 mg; I (E 2) (from $Ca(I_2O_3)_2$) 0.75 mg; Cu (E 4) (from $CuSO_4 \cdot 5H_2O$) 6 mg; Mn (E 5) (from MnO) 60 mg; Zn (E 6) (from ZnO) 110 mg; Se (E 8) (from Na_2SeO_3) 0.37 mg.

[3] Natugrain TS.

[4] Natuphos E 10000 G.

[5] Values in brackets include the expected nutrient contribution from Natuphos E 10000 G according to Table 2.

Supplementary Table S2. Composition of diets for the grower and finisher phases of trial 1.

	**Grower-1 (43-74 days)**			**Grower-2 (74-112 days)**			**Finisher (112-144 days)**		
	**T1 Positive Control (MCP)**	**T2 No MCP + Phytase (Red. Matrix)**	**T3 No MCP + Phytase (Ext. matrix)**	**T1 Positive Control (MCP)**	**T2 No MCP + Phytase (Red. Matrix)**	**T3 No MCP + Phytase (Ext. matrix)**	**T1 Positive Control (MCP)**	**T2 No MCP + Phytase (Red. Matrix)**	**T3 No MCP + Phytase (Ext. matrix)**
Ingredients (g/kg feed)									
Wheat	431.5	448.2	467.4	467.3	478.1	493.8	204.6	208.7	223.4
Barley	250	250	250	200	200	200	460	460	460
Soybean meal (48% CP)	107.5	105.8	101.6	67.4	67.9	63.1	33.6	37.8	30.7
Rapeseed meal	75	75	75	130	130	130	180	180	180
Sunflower meal (37% CP)	70	70	70	70	70	70	50	50	50
Animal fat	40.3	34.2	19.4	43.8	39.3	28.4	54.4	51.1	43.4
L-Lysine-HCl	2.93	2.92	2.8	1.86	1.81	1.77	0.95	0.79	0.88
L-Threonine	0.53	0.51	0.42	-	-	-	-	-	-
DL-Methionine	0.02	0.002	-	-	-	-	-	-	-
Salt	4.23	4.24	4.24	3.96	3.96	3.96	3.65	3.65	3.66
Calcium carbonate	6.43	4.7	4.7	5.95	4.67	4.68	4.49	3.55	3.59
Monocalcium phosphate	7.34	-	-	5.42	-	-	3.89	-	-
Noxyfeed [1]	0.2	0.2	0.2	0.2	0.2	0.2	0.2	0.2	0.2
Vit-Min complex [2]	4	4	4	4	4	4	4	4	4
Carbohydrase (TXU-TGU/kg) [3]	560-250	560-250	560-250	560-250	560-250	560-250	560-250	560-250	560-250
Phytase (FTU/kg) [4]	-	1000	1000	-	500	500	-	300	300
Estimated nutrients (g/kg feed)									
Crude Protein	171	171.8	171.5 (175.3)	170.2	171.4	170.6 (173.4)	162.7	164.9	163.1 (165.1)
Crude Fibre	47.4	47.7	48	50.4	50.7	50.9	56.8	57.1	57.1
Fat	58.3	52.5	38	62	57.7	47	73	69.9	62.3
Ash	49.7	40.9	40.9	47.6	41.2	41.1	46.6	42.1	42
Energy (kcal ME/kg)	3180	3180	3173 (3180)	3175	3175	3170 (3175)	3150	3150	3146 (3150)
SID Lysine	8.9	8.9	7.75 (8.90)	7.7	7.7	7.59 (7.70)	6.3	6.3	6.22 (6.30)
SID Threonine	5.79	5.79	5.70 (5.79)	5.2	5.24	5.22 (5.29)	4.66	4.74	4.67 (4.72)
SID Methionine	2.76	2.76	2.76 (2.81)	2.83	2.85	2.85 (2.88)	2.57	2.6	2.59 (2.61)
SID Met+Cys	5.91	5.94	5.96 (6.08)	6.11	6.17	6.18 (6.26)	5.56	5.63	5.61 (5.66)
SID Tryptophan	1.94	1.95	1.95 (2.01)	1.9	1.92	1.92 (1.96)	1.64	1.67	1.65 (1.68)
Total calcium	6.7	4.80 (6.70)	4.80 (6.70)	6.5	5.10 (6.50)	5.10 (6.50)	5.9	4.90 (5.90)	4.90 (5.90)
Total phosphorous	6.05	4.43 (6.33)	4.46 (6.36)	5.94	4.74 (6.14)	4.76 (6.16)	5.81	4.97 (5.97)	4.97 (5.97)
Digestible P	2.8	1.28 (2.80)	1.29 (2.81)	2.5	1.38 (2.50)	1.39 (2.51)	2.3	1.50 (2.30)	1.50 (2.30)
Phytate P	2.81	2.84	2.87	3.02	3.05	3.06	3.02	3.05	3.05
Analysed nutrients (g/kg feed)									
Dry Matter	904	903	904	906	906	903	906	904	904
Crude Protein	167	166	168	186	185	179	169	174	171
Crude Fibre	46	42.5	42.3	50	30	47.1	52.4	52	52.5
Fat	55.9	50.4	36.8	60.5	57	47	72.1	67.6	60
Ash	47	42.5	41.6	44.7	38.4	38.6	43.4	41.4	40.1
Energy (kcal GE/kg)	4156	4156	4071	4209	4220	4169	4277	4284	4247

Total calcium	6.0	4.1	4.6	5.7	4.1	4.6	5.5	4.2	4.2
Total phosphorous	5.4	4.2	4.4	5.8	4.4	4.5	5.2	4.6	4.7
Phytate P	2.55	2.57	2.59	2.49	2.34	2.32	2.45	2.45	2.41
Phytase (FTU/kg)	-	1017	957	-	432	430	-	398	426
% recovery	-	102	96	-	86	86	-	133	142

[1] ITPSA, Barcelona, Spain. Contains BHT+ propyl galate (56%) and citric acid (14%).

[2] Provides per kg feed: vitamin A (E 672) 5500 UI; vitamin D3 (E 671) 1100 UI; vitamin E (alfa tocopherol) 25 mg; vitamin B1 0.5 mg; vitamin B2 1.4 mg; vitamin B6 1 mg; vitamin B12 8 μg; vitamin K3 0.5 mg; calcium panthotenate 5.6 mg; nicotinic acid 8 mg; choline 120 mg; Fe (E 1) (from FeSO4·7H2O) 80 mg; I (E 2) (from Ca(I2O3)2) 0.5 mg; Co (E 3) (from 2CoCO3·3Co(OH)2·H2O) 0.4 mg; Cu (E 4) (from CuSO4·5H2O) 5 mg; Cu (E 4) (from aminoacids quelate) 5 mg; Mn (E 5) (from MnO) 40 mg; Zn (E 6) (from ZnO) 100 mg; Se (E 8) (from Na2SeO3) 0.25 mg.

[3] Natugrain TS.

[4] Natuphos E 10000 G.

Values in brackets include the expected nutrient contribution from Natuphos E 10000 G according to Table 2.

Supplementary Table S3. Composition of diets for the pre-starter and starter phases of trial 2.

	Pre-starter (0-14 days)			Starter (14-41 days)		
	**T1 Positive Control (MCP)**	**T2 No MCP + Phytase (Red. Matrix)**	**T3 No MCP + Phytase (Ext. matrix)**	**T1 Positive Control (MCP)**	**T2 No MCP + Phytase (Red. Matrix)**	**T3 No MCP + Phytase (Ext. matrix)**
			Ingredients (g/kg feed)			
Maize	90.3	110	132.8	255.3	274.7	292.1
Wheat	-	-	-	100	100	100
Extr. Maize:Wheat 60:40	250	250	250	-	-	-
Barley	210	210	210	180	180	180
Sweet milk whey	84	84	84	65	65	65
Dextrose	30	30	30	-	-	-
Spray dried plasma	55	55	55	-	-	-
Soybean meal (48% CP)	111.2	108.4	101.1	181.1	178.6	177
Rapeseed meal	52.5	52.5	52.5	70	70	70
Sunflower meal (37% CP)	52.5	52.5	52.5	60	60	60
Animal fat	31.7	24.5	9.1	50.4	43.4	28.1
L-Lysine-HCl	3.7	3.8	3.8	4.9	4.9	4.8
L-Threonine	1.1	1.1	1.1	1.9	1.9	1.8
DL-Methionine	1.2	1.2	1.1	1.4	1.4	1.2
L-Tryptophan	0.3	0.3	0.2	0.5	0.5	0.4
L-Valine	-	-	-	0.6	0.6	0.5
Salt	-	-	-	2.3	2.3	2.3
Calcium carbonate	6	4.1	4.2	6.2	4.2	4.2
Monocalcium phosphate	8.2	0	0	8	0	0
Noxyfeed [1]	0.2	0.2	0.2	0.2	0.2	0.2
Vit-Min complex [2]	4	4	4	4	4	4
Amasil NA	8	8	8	8	8	8
Carbohydrase (TXU-TGU/kg) [3]	560-250	560-250	560-250	560-250	560-250	560-250
Phytase (FTU/kg) [4]	-	3000	3000	-	2000	2000
			Estimated nutrients (g/kg feed)			
Crude Protein	197.5	197.4	195.2 (199.5)	190	190	189.8 (194.1)
Crude Fibre	37.5	37.8	38	41.9	42.2	42.5
Fat	50.6	44.1	29.4	69.1	62.7	48
Ash	53	43	42.8	53.6	43.7	43.8
Energy (kcal ME/kg)	3285	3285	3214 (3285)	3280	3280	3209 (3280)
SID Lysine	12.8	12.8	12.63 (12.80)	12	12	11.83 (12.00)
SID Threonine	8.32	8.32	8.21 (8.32)	7.8	7.8	7.69 (7.80)
SID Methionine	3.84	3.84	3.79 (3.84)	4.16	4.15	4.02 (4.07)
SID Met+Cys	7.72	7.72	7.65 (7.77)	7.08	7.08	6.96 (7.08)
SID Tryptophan	2.56	2.56	2.50 (2.56)	2.4	2.4	2.33 (2.40)
Total calcium	7	4.85 (7.00)	4.85 (7.00)	7.3	5.15 (7.30)	5.15 (7.30)
Total phosphorous	6.68	4.85 (7.00)	4.86 (7.01)	6.46	4.66 (6.81)	4.70 (6.85)
Digestible P	3.8	2.10 (3.82)	2.10 (3.82)	3.3	1.58 (3.30)	1.64 (3.36)
Phytate P	2.32	2.35	2.37	2.75	2.77	2.8
			Analysed nutrients (g/kg feed)			
Dry Matter	892	895	922	897	897	898

Crude Protein	200	206	214	213	207	192
Crude Fibre	38.7	35.9	34.1	36	37.1	37.2
Fat	41.6	35.7	26.8	59.5	55.9	43.3
Ash	48.5	43.1	44.3	51.9	40.2	37.2
Energy (kcal GE/kg)	4047	4072	4091	4193	4224	4131
Total calcium	6.43	4.45	4.74	7.16	4.91	4.68
Total phosphorous	6.66	4.97	5.08	6.56	4.63	4.48
Phytate P	2.19	2.23	2.27	2.51	2.52	2.52
Phytase (FTU/kg)	-	2908	1178	-	1996	1913
% recovery	-	97	39	-	100	96

[1] ITPSA, Barcelona, Spain. Contains BHT+ propyl galate (56%) and citric acid (14%).
[2] Provides per kg feed: vitamin A (E 672) 10000 UI; vitamin D3 (E 671) 2000 UI; vitamin E (alfa tocopherol) 25 mg; vitamin B1 1.5 mg; vitamin B2 3.5 mg; vitamin B6 2.4 mg; vitamin B12 20 µg; vitamin K3 1.5 mg; calcium panthotenate 14 mg; nicotinic acid 20 mg; folic acid 0.5 mg; biotin 50 µg; Fe (E 1) (from FeSO4·H2O) 120 mg; I (E 2) (from Ca(I2O3)2) 0.75 mg; Cu (E 4) (from CuSO4·5H2O) 6 mg; Mn (E 5) (from MnO) 60 mg; Zn (E 6) (from ZnO) 110 mg; Se (E 8) (from Na2SeO3) 0.37 mg.
[3] Natugrain TS.
[4] Natuphos E 10000 G.
Values in brackets include the expected nutrient contribution from Natuphos E 10000 G according to Table 2.

Supplementary Table S4. Composition of diets for the grower and finisher phases of trial 2.

	Grower-1 (41-71 days)			Grower-2 (71-116 days)			Finisher (116-138 days)		
	**T1 Positive Control (MCP)**	**T2 No MCP + Phytase (Red. Matrix)**	**T3 No MCP + Phytase (Ext. matrix)**	**T1 Positive Control (MCP)**	**T2 No MCP + Phytase (Red. Matrix)**	**T3 No MCP + Phytase (Ext. matrix)**	**T1 Positive Control (MCP)**	**T2 No MCP + Phytase (Red. Matrix)**	**T3 No MCP + Phytase (Ext. matrix)**
Ingredients (g/kg feed)									
Maize	100	100	100	47	58.8	72.8	115	115	115
Barley	567.7	583.6	602.3	605	605	605	639.3	651.8	666.4
Soybean meal (48% CP)	102.8	100.8	96.2	59	58.2	55.6	53.6	50.8	46.4
Rapeseed meal	75	75	75	130	130	130	100	100	100
Sunflower meal (37% CP)	70	70	70	70	70	70	40	40	40
Animal fat	58.2	53.1	39.2	67.4	62.8	51.6	51.7	48.1	38
L-Lysine-HCl	3.2	3.2	3.1	2.1	2.2	2.1	1.9	1.9	1.9
L-Threonine	0.8	0.8	0.7	0.1	0.1	0.1	0.1	0.1	0.1
DL-Methionine	0.2	0.2	0.1	-	-	-	-	-	-
Salt	4.2	4.2	4.2	3.9	3.9	3.9	3.7	3.7	3.7
Calcium carbonate	6.4	4.7	4.7	6	4.6	4.7	5.6	4.2	4.2
Monocalcium phosphate	7.1	0	0	5.1	0	0	4.8	0	0
Noxyfeed [1]	0.2	0.2	0.2	0.2	0.2	0.2	0.2	0.2	0.2
Vit-Min complex [2]	4	4	4	4	4	4	4	4	4
Carbohydrase (TXU-TGU/kg) [3]	560-250	560-250	560-250	560-250	560-250	560-250	560-250	560-250	560-250
Phytase (FTU/kg) [4]	-	1000	1000	-	500	500	-	500	500
Estimated nutrients (g/kg feed)									
Crude Protein	165.9	166.5	166.1 (169.9)	166	166.3	165.8 (168.6)	140	140	139.3 (142.1)
Crude Fibre	54.8	55.4	56.2	60.4	60.6	60.8	53	53.4	53.9
Fat	77	72.1	58.6	85.9	81.7	70.9	71.8	68.5	58.5
Ash	51.7	43.2	43.4	50.3	44	44	45.5	39.4	39.5
Energy (kcal ME/kg)	3180	3180	3110 (3180)	3175	3175	3123 (3175)	3150	3150	3098 (3150)
SID Lysine	8.9	8.9	7.75 (8.90)	7.7	7.7	7.59 (7.70)	6.3	6.3	6.19 (6.30)
SID Threonine	5.79	5.79	5.69 (5.79)	5.01	5.01	4.94 (5.01)	4.16	4.16	4.09 (4.16)
SID Methionine	2.76	2.76	2.71 (2.76)	2.63	2.63	2.63 (2.67)	2.2	2.2	2.20 (2.23)
SID Met+Cys	5.59	5.61	5.58 (5.68)	5.59	5.6	5.60 (5.68)	4.81	4.82	4.82 (4.89)
SID Tryptophan	1.72	1.73	1.72 (1.78)	1.69	1.69	1.68 (1.72)	1.4	1.4	1.39 (1.43)
Total calcium	6.7	4.80 (6.70)	4.80 (6.70)	6.5	5.10 (6.50)	5.10 (6.50)	5.9	4.00 (5.90)	4.00 (5.90)
Total phosphorous	6.06	4.48 (6.38)	4.52 (6.42)	5.95	4.67 (6.07)	4.83 (6.23)	5.35	4.28 (5.68)	4.30 (5.70)
Digestible P	2.8	1.33 (2.85)	1.33 (2.85)	2.5	1.38 (2.50)	1.44 (2.56)	2.3	1.30 (2.42)	1.31 (2.43)
Phytate P	2.79	2.82	2.83	2.97	2.99	3.01	2.6	2.61	2.62
Analysed nutrients (g/kg feed)									
Dry Matter	905	901	903	903	905	905	910	911	906
Crude Protein	169	165	165	161	162	161	141	137	141
Crude Fibre	52.3	53.5	53.6	58.9	58.7	58	51.3	54	53.9
Fat	68.9	62.1	50.9	78	72.7	63.5	65.9	62.5	53
Ash	55.3	44.1	46.6	45.6	36	36.8	37.2	31.2	34.2
Energy (kcal GE/kg)	4205	4195	4145	4278	4287	4224	4213	4192	4142

Total calcium	6.84	4.6	4.42	6.1	4.84	4.92	5.47	4.19	4.33
Total phosphorous	5.94	4.4	4.39	5.62	4.51	4.54	4.97	4.21	4.21
Phytate P	2.58	2.56	2.65	2.57	2.62	2.69	2.6	2.64	2.59
Phytase (FTU/kg)	-	950	985	-	486	635	-	636	501
% recovery	-	95	99	-	97	127	-	127	100

[1] ITPSA, Barcelona, Spain. Contains BHT+ propyl galate (56%) and citric acid (14%).

[2] Provides per kg feed: vitamin A (E 672) 5500 UI; vitamin D3 (E 671) 1100 UI; vitamin E (alfa tocopherol) 25 mg; vitamin B1 0.5 mg; vitamin B2 1.4 mg; vitamin B6 1 mg; vitamin B12 8 μg; vitamin K3 0.5 mg; calcium panthotenate 5.6 mg; nicotinic acid 8 mg; choline 120 mg; Fe (E 1) (from FeSO4·7H2O) 80 mg; I (E 2) (from Ca(I2O3)2) 0.5 mg; Co (E 3) (from 2CoCO3·3Co(OH)2·H2O) 0.4 mg; Cu (E 4) (from CuSO4·5H2O) 5 mg; Cu (E 4) (from aminoacids quelate) 5 mg; Mn (E 5) (from MnO) 40 mg; Zn (E 6) (from ZnO) 100 mg; Se (E 8) (from Na2SeO3) 0.25 mg.

[3] Natugrain TS.

[4] Natuphos E 10000 G.

Values in brackets include the expected nutrient contribution from Natuphos E 10000 G according to Table 2.
